# A Forensic Diagnostic Algorithm for Drug-Related Deaths: A Case Series

**DOI:** 10.3390/toxics10040152

**Published:** 2022-03-22

**Authors:** Antonina Argo, Stefania Zerbo, Roberto Buscemi, Claudia Trignano, Elisabetta Bertol, Giuseppe Davide Albano, Fabio Vaiano

**Affiliations:** 1 PROMISE Department, University of Palermo, 90100 Palermo, Italy; antonella.argo@unipa.it (A.A.); stefania.zerbo@unipa.it (S.Z.); rbuscem@hotmail.com (R.B.); 2 Department of Biomedical Sciences, University of Sassari, 7100 Sassari, Italy; claudia.trignano@uniss.it; 3 Department of Health Sciences, University of Florence, 50121 Florence, Italy; elisabetta.bertol@unifi.it (E.B.); fabio.vaiano@unifi.it (F.V.)

**Keywords:** drug-related deaths, forensic diagnosis, diagnostic algorithm, drug intoxication diagnosis

## Abstract

The best evidence provided in the literature worldwide suggests the importance of harmonizing the investigation in drug-related fatalities. In this study, the application of a multidisciplinary approach in eight cases of drug-related deaths is presented. Although death scene findings could be highly suggestive of drug intoxication, external examination and toxicological screening test alone are insufficient. There are several variables, and it is not always easy to give the proper interpretation of the drug detection. A complete autopsy is necessary to correctly complete organ and tissues sampling for further histological and toxicological studies and obtain body fluids. The use of peripheral blood is recommended to avoid artifacts. The collection of many specimens is warranted to get more responses. The sampling aims to provide a picture of the distribution of the substance in the body. The sample and the selection of the drugs and the matrices to investigate are case-dependent. The presented diagnostic algorithm provides the coroner with all the elements to investigate drug-related deaths and cooperate with toxicologists. Toxicological forensic diagnosis is still extremely heterogeneous in regional and national contexts. Funding for method development, research, networking, facilities, and technologies improvement is mandatory to standardize the toxicological investigation.

## 1. Introduction

Nowadays, deaths from overdose prevalence continue to increase also due to the diffusion of novel illicit drugs [1]. Moreover, a higher risk of overdose is associated with younger age, polydrug use, and heroin abuse [2,3]. Postmortem toxicology has a crucial role in determining the cause and manner of death when illicit drugs assumption or poisoning is suspected. Therefore, it has relevant public health and social function [4]. History and death scene investigation alone is not sufficient to predict a drug intoxication. The toxicology laboratory can identify and measure drugs in biological samples assisting the forensic pathologist and the medical examiner in their work [5,6]. The recommendations regarding the investigation, evaluation, and certification of deaths related to drugs misuse were recently updated by the National Association of Medical Examiners and an expert panel of medical toxicologists [1,7]. The best evidence provided in the literature worldwide suggests the importance of harmonizing the investigation in drug-related fatalities. The quality of forensic diagnosis is affected by the quality of toxicological analyses, technical equipment, personnel competence, financial resources. Moreover, there are extreme regional and national differences in post-mortem diagnosis of drug-related deaths. Such scenarios influence investigation results, leading to a lower quality contributing to epidemiological reports and public health and law enforcement measures [8,9,10,11]. In this regard, high-quality and standardized methodologies are required in forensic diagnosis in case of recognition of drug-related deaths When a drug intoxication is suspected, a multidisciplinary approach is mandatory to establish the cause and manner of death. This article reports eight cases of death related to drug misuse or abuse of substances in which a complete diagnostic algorithm based on the best evidence provided in the literature was essential to answering forensic questions.

## 2. Materials and Methods

### 2.1. Sample Collection

A retrospective analysis of the autopsy records of the drug-related deaths cases in which toxicological studies were performed between 2019 and 2021 in the Forensic Toxicology Unit of “AOU-Careggi” (Florence, Italy) was carried out. The cases with weak or missing information about social and medical history, death scene investigation, a complete autopsy, histological analyses, alcohol blood concentration testing, qualitative toxicological screening urine samples for a full toxicological panel, confirmation toxicological analyses for quantitative testing were excluded. Decomposed bodies were also excluded from the study. In all selected cases, death scene investigation, external examination, a complete autopsy, and histological examination were performed. Toxicological analysis in all cases involved blood alcohol concentration, toxicological panel for illicit drugs (opioids, ketamine, methadone, amphetamines, cocaine and metabolites, THC), gamma-hydroxybutyrate acid (GHB), benzodiazepines, barbiturates, and buprenorphine, by performing a qualitative test on urine samples (immunoenzymatic technique). Qualitative analysis was followed by a quantitative test with gas chromatography-mass spectrometry (GC-MS) and liquid chromatography with tandem mass spectrometry (LC-MS/MS). Blood, urine, and vitreous humor samples were obtained during the external examination. They were stored at −20 °C. Only autopsies with brain, lungs, heart, kidneys, liver, and spleen samples were collected. For further toxicological investigations, sections of the brain, liver, kidney, and bile were collected and stored at −20 °C. Hair samples were obtained in all cases, gastric content when an oral drug assumption was suspected. All selected autopsies were performed within four days after death was determined. All cadavers were stored at −4 °C. The specimen collection protocol was applied in the selected cases, and specimens were accompanied by a chain of custody form [1]. The manuscript describes a series of human studies performed in charge of the Prosecutor’s Office for forensic purposes; therefore, ethics committee or institutional review board approval is not required. All procedures performed in this study were in accordance with the ethical standards of the institution and with the 1964 Helsinki Declaration and its later amendments or comparable ethical standards. Informed consent was obtained from the relatives by Prosecutor Office. Cases that did not meet inclusion criteria were excluded. From the analysis of death scene investigations, autopsy reports, toxicological analyses, and the information gathered from the police, eight significant explanatory cases of deaths related to the administration of drugs or abuse of substances were included.

### 2.2. Histological Analysis

A routine microscopic histopathological study was performed using hematoxylin-eosin (H&E) staining. Specimens from organs were fixed in 10% buffered formalin. After an overnight wash, samples were dehydrated in graded ethanol cleared in xylene and paraffinembedded. Tissue paraffin blocks were then cut (4 µm thickness) using a microtome, and sections were mounted on silane-coated slides (Dako, Glostrup, Denmark) and stored at room temperature. Sections then were stained with H&E and observed using a Zeiss Axioplan light microscope (Carl Zeiss, Oberkochen, Germany) for morphological examination.

### 2.3. Toxicological Analysis

#### 2.3.1. EMIT^®^ Immunoassay Qualitative Screening Test

The most frequent drugs of abuse (amphetamines, THC, cocaine, methadone, and opioids) and medically prescribed drugs (barbiturates, benzodiazepines, antidepressants, anticonvulsants) were analyzed by performing a screening test on urine samples with an EMIT^®^ Siemens VIVA-E drug testing system (Siemens, Newark, DE, USA), according to the manufacturer instructions [12].

#### 2.3.2. Alcohol Level Determination: Head Space-Gas Chromatography (HS-GC)

Our laboratory procedure was applied to measure blood alcohol content [13]. HS-GC analysis was performed in a 1 mL blood sample aliquot added in a vial with a water solution with 1 g/L concentration of the internal standard (2-butanol) and 30 mg of sodium fluoride.

#### 2.3.3. Amphetamines, Cocaine, Methadone and Opioids

Simultaneous extraction of basic organic nitrogenous substances was performed on 2–4 mL of samples. Each sample was added with internal standards (bupivacaine 1.000 ng/mL, scopolamine 1.000 ng/mL, nalorphine 1.000 ng/mL, and pinazepam 100 ng/mL). The samples were agitated for 1 min and then centrifuged at 4500 rpm for 5 min. A saturated solution of ammonium sulphate and concentrated hydrochloric acid was added. The filtrate obtained was added with cyclohexane and centrifuged; the washing phase was alkalized with ammonia up to pH 7.5 and extracted with ethyl acetate. After solvent and residue evaporation, after acetone use, 1 µL of the mixture was then injected in the GC-MS system with SCAN acquisition mode for alkaloid nitrogenous organic substances searching. For opioids extraction, the washing phase was treated with ammonia and then with a mixture of chloroform and isopropyl alcohol (3 to 1 ratio) until reaching pH 8.5. The extract collected, derivatized with 50 µL of BSTFA at 70 °C for 30 min, was analyzed in GC-MS with SIM (selected ion monitoring) acquisition mode [14].

#### 2.3.4. Tetra-Hydro-Cannabinol (THC)

The extraction methodology was the same as in a previous paper [13]. Biological samples (500 µL) were added with 1 mL of potassium hydroxide 10 M and kept at 60 °C for 15 min. One microliter of volume solution was injected into the GC-MS system for the analysis.

#### 2.3.5. Gamma-Hydroxybutyrate Acid (GHB)

GHB determination was performed following the standard procedure in our laboratory [15]. Briefly, 100 µL of biological fluid was added by 50 µL of GHB-D6 (10 ng/µL, IS) and liquid–liquid extracted by 500 µL of ethyl acetate. The supernatant was dried under an N2 stream at 45 °C and the residue was derivatized with 75 µL of BSTFA 1% TMCS at 90 °C for 5 min. One microliter of the mixture was then injected into the GC-MS system.

#### 2.3.6. GC–MS Instrumentation

The GC–MS system consisted of an Agilent 7890A GC system equipped with an Agilent 7683B series autosampler and interfaced via an electronic impact source to a single quadrupole Agilent 5975C mass spectrometer. Helium was used as a gas carrier at a 1 mL/min constant flow. The GC-MS methodology was the same used in previous studies [13].

#### 2.3.7. LC-MS/MS for 120 New Psychoactive Substances (NPS), Benzodiazepines and Antidepressants

A 200 µL aliquot of biological fluid was added with 700 µL of acetonitrile at 0 °C. The supernatant was dried under an N2 stream and reconstituted in 100 µL of water. Six microliters of the mixture was injected into the LC-MS/MS system. The analysis was conducted using an HPLC Agilent 1290 Infinity system coupled via electrospray ion (ESI) source to an Agilent 6460 Triple Quad LC/MS. The source parameters were: gas temperature 325 °C; gas flow rate 10 L/min; nebulizer 20 psi; capillary 4000 V. Chromatographic separations were carried out on a Zorbax Eclipse Plus C18 column (2.1 mm × 1000 mm, 1.8 µm, Agilent Technologies, Santa Clara, CA, US). Elution gradients and multiple reaction monitoring transitions for all the detectable substances are described in the previous paper [16].

## 3. Results

Table 1 shows a summary of clinical and social history, external examination, autopsy findings, and the cause and manner of death.

### 3.1. Case 1

A 52-year-old man with a positive history of opioid and cocaine assumption was found naked in his bedroom with no vital signs. Metal cooker spoons and knives, a broken glass bottle, and burn signs were found inside a bedside table drawer. Several benzodiazepine vials with lormetazepam were found in the trash can, as well as empty beer bottles and cigarettes. Multiple abrasions in the face were present, no injection marks were observed. A postmortem total body CT scan was performed to exclude the presence of traumatic lesions.

Liquid material in the lower airways and lung edema was observed in postmortem CT scans. In situ observation, upper and lower airways showed abundant liquid reddish material with foam. Histopathological analyses showed diffuse endoalveolar hemorrhagic edema and acute emphysema in lung samples and diffuse stasis in all organ samples. A qualitative screening test on urine samples with the immunoenzymatic technique was positive for opioids and cocaine metabolites. The alcohol concentration in blood was 0.44 g/L. Quantitative testing by gas chromatography-mass spectrometry (GC-MS) was performed in urine and blood samples. Codeine, morphine, 6-acetyl morphine (6 AM), cocaine metabolites, and lormetazepam were detected in blood and urine samples (Table 2). Death was attributed to central respiratory failure due to benzodiazepines and opioids’ recent assumption with the previous co-assumption of cocaine and alcohol. The manner of death was accidental.

### 3.2. Case 2

A 41-year-old man with a positive history of illicit drugs assumption was found decedent in his car. He had been discharged two days before from a recovery center for drug abusers. The car was closed, and there were no intrusion signs. During the death scene investigation, two empty syringes were found under the car seat and taken by local law enforcement. External examination showed two recent injection marks in the right arm. No signs of aggression or trauma were detected. Femoral vein blood and urine samples were taken during the external examination. A white/reddish foam in the upper airways and cerebral edema was present. Injection mark skin samples were taken during autopsy for histological analysis. Hemorrhagic alveolar edema acute and chronic emphysema were present in lung samples. Petechial hemorrhages were observed in lung and heart samples. Injection marks skin samples showed hemorrhage in interstitial space, dermal and subdermal layer. A screening test on urine samples with the immunoenzymatic technique was positive for opioids, cocaine, and THC metabolites. Quantitative testing by GC-MS was performed in urine and blood samples. Cocaine metabolites were present in blood and urine. Codeine, morphine, 6 AM, and THC were detected in urine as signs of recent and previous death assumptions (Table 2). Death was attributed to respiratory failure due to co-assumption (“speedball”) of cocaine and opioids. The manner of death was accidental.

### 3.3. Case 3

A 66-year-old woman was found in her bedroom with a plastic bed covering her face. She had a positive medical history of depression, psychiatric disorders, and alcoholism. She had attempted suicide several times. Benzodiazepines drops and pills were present nearby her bed. The house was tidy and cleaned, and there were no signs of intrusion. The external examination excluded any remarkable sign of trauma and/or aggression. Femoral vein blood and urine samples were taken during the external examination. Acute emphysema and edema were present in lung samples. Stasis and edema were observed in brain samples. The alcohol concentration in blood was 1.01 g/L. A qualitative screening test on urine samples with the immunoenzymatic technique was negative for illicit drugs and positive for benzodiazepines testing. Quantitative testing by GC-MS was performed in urine, blood, and gastric content samples and was negative for opioids, cocaine, THC, and all main illicit drugs metabolites. High concentrations of lormetazepam were found in blood, urine, and gastric content (Table 2). The cause of death was suffocation asphyxia after the assumption of alcohol and benzodiazepines. Suicide was the manner of death.

### 3.4. Case 4

A 21-year-old man was found in his bedroom by his father with white foam coming out from the mouth. Emergency Services attempted Cardiorespiratory resuscitation which was unsuccessful. External examination showed multiple injection marks in the upper arms. The autopsy excluded evidence of trauma and showed brain and lung edema. Quantitative testing by GC-MS was performed in urine, blood, liver, bile, and brain samples. A qualitative screening test on urine samples was positive for opioids, cocaine, and cannabis. Opioids and cocaine metabolites were detected in blood, urine, liver, brain, and bile samples after the GC-MS test. Cocaine concentrations were not relevant for death determination (Table 2). Death was attributed to cardiorespiratory failure due to acute opioid intoxication (intravenous use) with concomitant cocaine assumption. Manner of death was accidental.

### 3.5. Case 5

A 16-year-old woman was found in her house by her boyfriend with no life signs. The decedent and her father had a positive history of illicit drugs assumption and were followed by local social services. Her father kept methadone vials in the house. External examination showed injection marks in the right and left elbow. The autopsy excluded traumatic lesions. Diffuse stasis, endoalveolar edema, acute emphysema, and myocardial fibrosis were found. The screening urine test was positive for cocaine, methadone, THC, and opioid metabolites. GC-MS was used to search for cocaine, methadone, THC, and opioid metabolites in blood, urine, brain, liver samples. Quantitative testing showed very high concentrations of methadone in the blood. Methadone, cocaine, and opioid metabolites were detected in all the examined samples (Table 2). The cause of death was acute respiratory failure due to a methadone overdose powered by acute heroin intoxication and co-assumption of cocaine. The manner of death was accidental.

### 3.6. Case 6

A 54-year-oldmale inmate with a positive history of cocaine, heroin, and methadone abuse was found with no life signs inside his cell. He had a positive history of previous suicide attempts and was followed for rehab by local social services. He underwent therapy with methadone and anxiolytics. External examination showed lips and hand nails cyanosis. The autopsy showed diffuse stasis lung edema with foam coming out from the lungs after compression. Hemorrhagic petechiae were observed in the organs. A screening urine toxicological test was positive for methadone. A GC-MS test was performed for quantitative analysis in liver, brain, blood, urine, and bile samples. Methadone metabolites were detected in all samples. Methadone and opioid metabolites were found in hair samples as a sign of previous use (rehab therapy) (Table 2). Death was attributed to a respiratory failure due to a methadone overdose. The manner of death was accidental.

### 3.7. Case 7

A 53-year-old man with a positive history of illicit drugs and alcohol abuse was found decedent in his house nearby his kitchen table. Death scene examination showed two syringes with blood, paper boxes, and drugs vials on the table and no evidence of intrusion and aggression. External examination showed two bloody injection marks in the elbows and excluded trauma. The autopsy showed foam and reddish material in the lungs and lower airways. Brain and pulmonary edema were observed during microscopic examination. The qualitative screening toxicological test in urine samples was positive for opioids and cocaine metabolites (benzoylecgonine). GC-MS quantitative testing in liver, brain, blood, urine, and bile samples showed high concentrations of cocaine, benzoylecgonine, and morphine. Cocaine, benzoylecgonine, and morphine were detected in hair also, suggesting a chronic and previous abuse (Table 2). The cause of death was acute cocaine intoxication (endovenous use) with a recent assumption of morphine. The manner of death was accidental.

### 3.8. Case 8

A 25-year-old man with a positive history of occasional heroin and anxiolytics was found decedent on his bed. Emergency services’ resuscitative attempts were unsuccessful. Death scene examination showed vomit on the pillow and no evidence of trauma. During the external examination, two recent injection marks were detected. After compression, the autopsy showed diffuse stasis, foam, and blood coming out from the lungs. Abundant alveolar edema was observed. The qualitative screening toxicological test inurine samples was positive for opioids, benzodiazepine metabolites, and THC. Opioids (codeine, morphine, 6-acetyl morphine) were present in blood, urine, liver, brain, and bile samples after GC-MS. GC-MS of blood showed positive results for diazepam and delorazepam. Blood alcohol concentration was 0.44 g/L. Opioids were found in hair after GC-MS. Blood benzodiazepines concentrations were in the therapeutic range. Positive results for opioids in hair suggest previous assumptions (Table 2). The cause of death was respiratory failure due to an acute opioid intoxication (endovenous use). The manner of death was accidental.

## 4. Discussion

**Figure 1:**
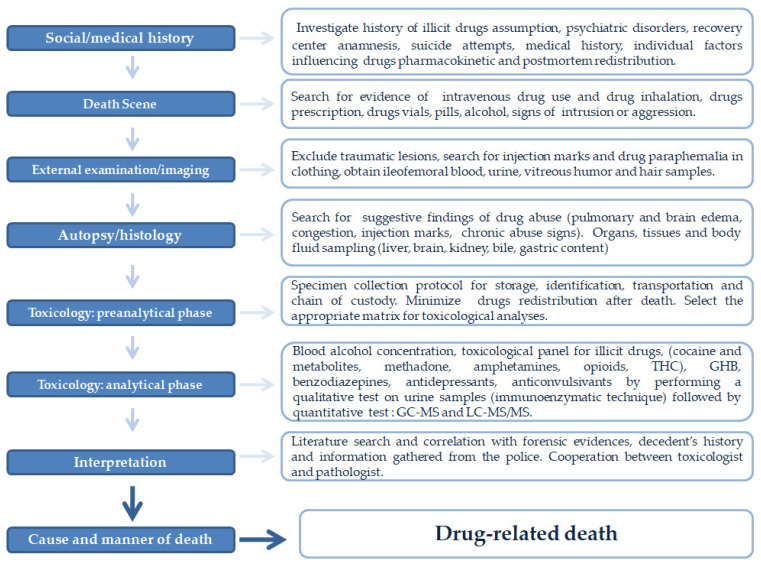
A diagnostic algorithm for managing suspected deaths related to the administration of drugs or abuse of substances.

The forensic diagnosis of deaths related to drug intoxication requires a multidisciplinary method. Applying a diagnostic algorithm (Figure 1) based on the most recent evidence in the literature is crucial to establish the cause and manner of death and answer the forensic questions. Social and medical history, investigative information, death scene investigation, external examination, postmortem imaging, autopsy, histopathology, and toxicological analyses are part of a puzzle that must be assembled appropriately in forensic practice. Death scene investigation, history analysis, and screening tests alone offer incomplete evidence and a lack of confirmation for establishing a cause of death [6,17]. Screening tests (i.e., immunoassays) are characterized by low specificity and sensitivity. In this regard, gas chromatography-mass spectrometry (GC-MS) systems are a widespread technique for general unknown analyses [12]. However, GC-MS has some limitations. Its use is limited to thermostable compounds, and derivatization phases require more prolonged sample treatment procedures. In this regard, LC-MS/MS is a less demanding method for sample preparation. The derivatization step is not needed even if it can improve the ionization efficiency (IE). The low IE of some compounds and unavailability of mass spectra libraries are the main issues concerning LC-MS/MS applications. In our diagnostic algorithm, the LC-MS/MS screening method for general unknown analysis was focused on NPS, benzodiazepines, and other antidepressants.

Several factors in the death scene need to be taken into consideration to suggest a fatality related to drug misuse and a drug intoxication: social and medical history, positive history of illicit drugs assumption, evidence of intravenous drug use (needles, syringes, cooker spoons, tourniquet, transdermal patches, other drug paraphernalia), proof of other illegal drug or substance use, drugs prescriptions, pills, drops, empty drug vials nearby the cadaver, injection sites, and evidence of insufflation [4,18,19]. Circumstances of death need to be thoroughly investigated in orderto suggest a drug misuse:witness statements, previous medical history, illicit drug or alcohol use history, previous imprisonment, family history blood-borne virus status (HIV, hepatitis virus) [11].Furthermore, when a drug intoxication is suspected, several factors need to be studied in deep that may influence the drug pharmacokinetic: weight, physical activity, nutritional state, diseases, smoking, concurrent pharmacological therapy. In all presented cases, death scene findings were pivotal for the following investigations and highly suggestive of a drug-related fatality. These findings, the witness’s recounting, and terminal circumstances may immediately suggest a drug intoxication, point out further studies and guide toxicological analyses.

Although death scene findings could be highly suggestive of drug intoxication, external examination alone is less accurate than autopsy, especially when drugs are present in the decedent. The external examination has the role of excluding the presence of signs of aggression or trauma and of looking for an indication of illicit drugs abuse, such as injection marks, needle tracks, or any drug evidence or paraphernalia in the decedent’s clothing. Blood and urine samples need to be obtained as soon as possible because of the postmortem changes of the body and the redistribution of drugs. The best source of blood is the iliofemoral vein. Indeed, blood sampling before opening the body and as soon as possible minimize redistribution and contamination with other body fluids. If it isnot available, it is recommended to obtain blood from the subclavian vein, the heart or aorta, or any other intact vessel in decreasing order of desirability [4].

Nowadays, forensic imaging still has a marginal role in drug-related deaths diagnoses. Most of the studies in the literature showed no distinctive features of postmortem CT to establish the cause and manner of death in such cases. Cerebral edema, pulmonary edema, bladder distension, liquid material in the airways are frequent findings in postmortem CT but are not sensitive for death due to illicit drug intoxication. For this reason, the use of postmortem CT is not yet a routine in forensic practice [20,21,22]. In case 1, multiple abrasions were observed during an external examination, and postmortem CT allowed to exclude signs of trauma and aggression previous autopsy. Access to appropriate post-mortem imaging facilities is heterogeneous and limited around the world.Its use could be helpful and needs to be assessedon a case-by-case basis [11].In this regard, if available, postmortem CT could be beneficial to exclude the presence of traumatic lesions when external examination and death scene may be suggestive of aggression or trauma.

A complete autopsy is a gold standard for determining the cause of death. In the presented cases, autopsy findings were not specific but suggestive for drug abuse. In most cases, there are no distinctive pathological findings indicating intoxication or poisoning. Congestion, pulmonary edema, liquid material and foam in the upper and lower airways, petechiae hemorrhages in the pleura, and pericardium are frequent macroscopic features in drug-related deaths. Still, they are not evocative of specific substance misuse. However, the autopsy is always helpful to provide information about the decedent’s medical history and further macroscopic evidence that may be relevant to establish the cause of death, such as visible signs of chronic drugs and/or intravenous drugs consumption. The autopsy is crucial to establish if a drug or toxin has increased the body’s susceptibility toany natural disease [11]. Moreover, the autopsy is necessary for a correct and complete organ sampling for further histological and toxicological studies and to obtain body fluids.

Acute and chronic drug assumption leads to several microscopic alterations that may be helpful to recognize and suspect drug-related deaths. Hemorrhagic protein-rich pulmonary edema, brain edema, diffuse stasis, and acute emphysema are frequent findings in drugs intoxication characterized by acute respiratory function impairment [23,24]. The skin samples of injection marks need to be sampled for histological examination. Abundant hemorrhage in the interstitial space, derma, and subdermal layers are signs of recent injection marks and need to be investigated. Moreover, some histological alterations indicate chronic drugs exposure and need to be considered in the cause of death differential diagnosis. Several pathological findings suggest chronic intravenous drug abuse: pulmonary granuloma, glomerulosclerosis, axonal damage and neurovascular complications, cardiac interstitial lymphomonocytic infiltrates. Furthermore, chronic cocaine abuse can lead to histopathological changes in the cardiovascular system: fibrosis, diffuse vessels wall thickening, and hypertrophy of smooth muscle cells [24,25]. These results are essential to suggest previous illicit drugs abuse.

Postmortem forensic toxicology is still a great challenge because of body decomposition and drug redistribution after death. Multiple preanalytic elements influence the specimen quality and the toxicological results [26,27,28]. Postmortem redistribution (PMR) refers to the changes in drug concentrations after death. It involves redistributing drugs into the blood from solid organs such as the lungs, liver, and myocardium. Drug properties such as volume of distribution, lipophilicity, and pKa are essential factors. Basic, highly lipophilic drugs are frequently involved in PMR. Examples include tricyclic antidepressants, digoxin, and amphetamines. Blood sampling sites can influence redistribution phenomena. Other environmental conditions may affect postmortem redistribution of drugs: initial concentration, bacterial invasion, temperature, hypostasis, time, and corpse position. Coroners, before sampling, must be aware of the substance redistribution after death when a drug intoxication is suspected. Correlation with laboratory data and any available antemortem or perimortem clinical information is necessary to render a reasonable opinion on the cause of death [29,30]. Therefore, the time between death and autopsy, the transportation and storage of the corpse until autopsy, and the specimen’s collection and storage need to adhere tohigh-quality and standardized protocols in forensic practice [27,28]. Specimens collection protocol requires correct identification of the biological sample (name, address, and telephone number of the coroner, time and place of collection, type, source, amount, or volume of the specimen) as well as a documented chain of custody. It is crucial to maintain the integrity and the identity of the samples from the collection to the analytical results. The preanalytic phase is essential and may influence toxicological effects.

For this reason, analytical results need to be interpreted together with the patient history and all other elements involved in postmortem examination [31]. Interpretation of postmortem toxicology requires detailed knowledge of the clinical pharmacology, pharmacokinetics, and the toxicology of the substance under investigation. In this regard, the cooperation between toxicologists and pathologists is essential to guarantee adequate sampling and proper sample storage and transportation. The selected eight fatalities showed that death scene, decedent history, external examination, autopsy, and histological analysis were suggestive of drug misuse and guided toxicological analytical testing. The presented diagnostic algorithm provides the coroner with all the elements to investigate drug-related deaths and cooperate with toxicologists. When a drug-related death is suspected, a full toxicological panel is required, qualitative and quantitative, including opioids, cocaine, THC, methadone, amphetamine, GHB, benzodiazepines, barbiturates, ethanol, buprenorphine, anticonvulsants, antidepressants, new psychoactive drugs becoming prevalent [4,32,33]. In the presented algorithm, qualitative analysis on urine samples with the immunoenzymatic technique and blood alcohol concentration testing was followed by a quantitative confirmation test by GC-MS and LC-MS/MS analysis for NPS, benzodiazepines, and antidepressants.

Alternative matrices can be selected in toxicological analyses for forensic purposes. Alternative biological matrices can provide additional information and advantages instead of blood and urine testing in several aspects such as sample collection, detection window, redistribution after death, and sample preparation/analysis complexity. In addition, these matrices can be collected and analyzed when blood and urine are not available. However, each of these alternative matrices has its characteristics, advantages, and limitations, which need to be considered [34] (Table 3).

In six reported cases, non-traditional (blood and urine) matrices were selected for toxicological testing. In five out of eight cases, a complex panel of drugs with multiple illicit drugs co-assumption was detected by blood analysis. As can be seen in cases 6, 7, and 8, hair sampling and analysis are crucial for investigating previous and chronic polydrug use. Furthermore, these cases highlight the importance of a thorough investigation of death and the necessity for toxicology to be pivotal in forensic diagnosis. In most cases, a polydrug assumption was detected. Several interactions are described in the literature among different drugs on vital functions that may have a role in death pathogenesis. The pathologist must be aware of the pharmacodynamics of illicit drugs and psychoactive substances to correctly interpret analytical results [35,36,37,38,39].

The selection of the specimen should be based on the types of expected substances. Many drugs show postmortem changes and instability. Cocaine and heroin are rapidlymetabolized. There are several variables, and it is not always easy to give the proper interpretation of the drug detection. The use of peripheral blood is recommended to avoid artifacts. The collection of many specimens is warranted to obtain more responses. The sampling aims to provide a picture of the distribution of the substance in the body. The sample and the selection of the substances and the matrices to investigate are case-dependent. It is still nebulous what can be defined as routine or appropriate in forensic toxicological analyses. Forensic pathologists’ and coroners’ choices must consider the facilities’ resources and the costs of investigations. The role of the toxicologist is to interpret the analyzedresults and needs to be supported by the best possible evidence and samples provided by the pathologist.

The presented diagnostic approach could be helpful in daily forensic practice when approaching drug-related deaths. However, one of the main limitations of forensic toxicological investigation is a missing standardization in regional and national contexts. Further efforts are needed to standardize toxicological testing for forensic purposes and improve technical developments of laboratory testing to improve laboratory capacity and meet high-quality and standardized requirements [8,9,10,29]. Postmortem concentrations must be interpreted according to all other findings of other forensic disciplines [27,33,40]. Moreover, all the conclusions collected must be related to the recent evidence provided by the literature to establish the cause and manner of death.Further efforts are needed to improve communication with other stakeholders (police, justice, health system), to undertake prevention and public health measures. In this regard, regional and national databases are needed to promote networking among toxicological laboratories [8].

## 5. Conclusions

In the selected cases, the application of the presented diagnostic algorithm highlights the necessity of a multidisciplinary approach and a complete and evidence-based methodology to establish the cause and manner of death in deaths related to the administration of drugs or abuse of substances.

The toxicology expertise is crucial to obtain adequate specimens for laboratory testing. In this regard, further studies and evidence-based guidelines are needed to implement sample collection, storage, and transportation protocols.

The methodology presented here was effective and reliable in assessing the cause and manner of death and can be applied in routine activity.

Drug-related deaths management requires the cooperation of many forensic disciplines and a mutual cross-talk between laboratory toxicologists and pathologists to support prosecutor activity.

Toxicological forensic diagnosis is still extremely heterogeneous in regional and national contexts. Funding for method development, research, networking, facilities, and technologies improvement is mandatory to standardize the toxicological investigation.

Drug misuse has been widespread and has increased in the last years, especially in the young population. Therefore, applying a standardized methodology in suspected drug-related fatalities is a relevant public health issue that must be vigorously promoted.

## Figures and Tables

**Table 1:** A summary of the main findings of the selected cases.

	Age Gender	History	Death Scene Investigation	External Examination/Imaging	Autopsy	Histopathology	Cause and Manner of Death
Case 1	52 y/o M	Opioid and cocaine abuse	Drugs paraphernalia in bedroom, lormetazepam vials in trash can	Multiple abrasions in the face; postmortem CT negative for trauma	Liquid reddish material in airways	Hemorrhagic pulmonary edema, stasis, acute emphysema	Central respiratory failure due to benzodiazepines and opioids assumption Accidental
Case 2	41 y/o M	Illicit drugs assumption; recently discharged from recovery	Two empty syringes under the car seat	Two injection marks in upper arm	Petachial hemorrhages in pleura	Hemorrhagic pulmonary edema, chronic and acute emphysema, recent injection marks	Respiratory failure due to co-assumption of cocaine and opioids Accidental
Case 3	66 y/o F	Previous suicide attempts; alcohol and antidepressants misuse	A plastic bag covering decedent face; alcohol and benzodiazepines nearby her bed; no signs of intrusion	No signs of trauma and or aggression	Liquid reddish material in airways	Hemorrhagic pulmonary edema, stasis, acute emphysema	Suffocation asphyxiaafterassumptionofalcoholandbenzodiazepines Suicide
Case 4	21 M	Nothing relevant	Injection marks, white foam in the mouth	No evidence of trauma	Stasis	Pulmonary and brain edema	Respiratory failure due to acute opioid intoxication with cocaine co-assumption
Case 5	16 y/o F	Illicit drugs assummpiton,followed by local social services	Methadone vials in the house	Injection marks	No evidence of trauma, diffuse stasis	Pulmonary edema, acute emphysema, myocardial fibrosis	Respiratory failure due to a methadone overdose powered by acute heroin intoxication
Case 6	54 y/o M	*Inmate*followed by local social services*.Previous suicide attempts and heroin, cocaine, and methadone assumption*	Found in his cell with no life signs	Cyanosis	No evidence of trauma, diffuse stasis, hemorrhagic petechiae	Pulmonary edema	Respiratory failure due to a methadone overdose Accidental
Case 7	53 y/o M	Illicit drugs and alcohol abuse	Drug paraphernalia (syringes, drug vials)	Injection marks	Stasis, pulmonary amd cardiac petechiae	Pulmonary and brain edema	Cardiorespiratory arrest due to acute cocaine intoxication (endovenous use) and recent assumption of opioids
Case 8	25 y/o M	Heroin and anxiolytics occasional use	Found in his bed with vomit on the pillow	Hand nails cyanosis, injection marks in the forearm	Foam and blood after lung compression, petechiae	Stasis, pulmonary and brain edema, blood in injection marks	Respiratory failure due to acute opioid intoxication (endovenous use)

**Table 2:** A summary of toxicological analysis results Eme: ecgonine methyl ester; Coca: cocaine; CE: cocaethylene; BE: benzoyl ecgonine; COD: codeine; 6-AM: 6 acetyl-morphine; MF: morphine; THC: tetrahydrocannabino; MT: methadone; EDDP: Ethylidene-1,5-dimethyl-3,3-diphenylpyrrolidine; ND: not detected).

	Urine Screening	Blood Alcohol g/L	Blood ng/mL	Urine (GS-MS) ng/mL	Liver (GS-MS) ng/g	Brain (GC-MS) ng/g	Other (Hair, Bile, Gastric Content)
Case 1	Opioids Cocaine Benzodiazepines	0.44	GS-MS Eme 57.41 Coca 48.73 CE 7.91 BE 1321.48 COD 1321.48 MF 126.83 6-AM 0.44 LC-MS/MS Lormetazepam 59.60	GS-MS Eme 106.01 Coca 6931.43 CE 372.48 BE 17,231.58 COD 66.99 MF 1606.14 6-AM 2.54 LC-MS/MS Lormetazepam 12,424.60	N.D.	N.D	N.D
Case 2	Opioids Cocaine THC	Negative	GS-MS EME 63.18 Coca 78.92 CE 0.91 BE 4457.18	GS-MS EME 129.68 Coca 9417.27 CE N.D BE 40,385.31 COD 130.79 MF 3490.51 6-AM 17.10 THC COOH 203.23	N.D.	N.D.	N.D.
Case 3	Benzodiazepines	1.01	LC-MS/MS Lormetazepam 56.73 Lorazepam 5.80 Alprazolam 85.94	LC-MS/MS Lormetazepam 1309.28 Lorazepam 157.81 Alprazolam 300.65	N.D.	N.D.	Gastric content(ng/mL) LC-MS/MS Lormetazepam 280.85 Lorazepam 6.08 Alprazolam 117.06
Case 4	Opioids Cocaine Cannabis	Negative	GS-MS Coca n.d. BE 489 COD 7 MF 25 6-AM 3	GS-MS Coca 3304 BE 14,227 COD 427 MF 9860 6-AM 3 THC 35	GS-MS Coca 24 BE 609 COD 14 MF 144 6-AM 2	GS-MS Coca 40 BE 154 COD 16 MF 54 6-AM 5	Bile (ng/mL) GS-MS Coca 3961 BE 594 COD 146 MF 6147 6-AM 21
Case 5	Opioids Methadone Cocaine Cannabis	Negative	GS-MS EDDP 81.46 MT 79.5 Coca Neg CE Neg BE 314 COD Neg MF 18 6-AM Neg	GS-MS EDDP 1412 MT 1550 Coca 3611 CE Neg BE 34 778 COD 279 MF 9928 6-AM 968	GS-MS EDDP 108.56 MT 117.56 Coca Neg CE Neg BE 532 COD Neg MF 103 6-AM Neg	GS-MS EDD 71.2 MT 57.1 Coca Neg CE Neg BE 216 COD Neg MF 55 6-AM Neg	N.D.
Case 6	Methadone	Negative	GS-MS EDDP 94 MT 1015	GS-MS EDDP 3778 MT 3415	GS-MS EDDP 122 MT 231	GS-MS EDDP 30 MT 630	Bile (ng/mL) GS-MS MDDP 1168 MT 614 Hair (ng/mg) GS-MS EDDP 0.133 MET 1.711 COD 0.093 MF 1.337 6-AM 0.605
Case 7	Opioids Cocaine	Negative	GS-MS Coca 18 BE 378 MF 230	GS-MS Coca 1319 BE 35513 MF2500	GS-MS Coca 25 BE 485 MF 800	GS-MS Coca 69 BE 71 MF 220	Bile (ng/mL) GS-MS Coca 3192 BE 1320 MF 12,000 Hair (ng/mg) GS-MS Coca 11.18 BE 2.48 MF 0.21
Case 8	Opioids Benzodiazepines	0.44	GS-MS COD Neg MF 106 6-AM 14 LC-MS/MS Diazepam 0.81 Delorazepam 0.40	GS-MS COD 146 MF 74.838 6-AM 57	GS-MS COD Neg MF 195 6-AM Neg	GS-MS COD Neg MF 64 6-AM 42	Bile (ng/mL) GS-MS COD 25 MF 44.396 6-AM 276 Hair (ng/mg) GS-MS COD 16 MF 219 6-AM 66

**Table 3:** Matrix properties, advantages, and limitations are summarized.

Matrix	Use	Advantages	Limitations
Blood	First choice specimen to detect, quantify and interpret substances/drugs concentratios	Best choice for acute intoxication or poisoning and quantitative data	Affected by postmortem redistribution after death, delayed collection after drug intake, putrefaction, patient diseases. No always easy to be collected (invasive). Short detection window
Urine	Standard method for screening qualitative test and general analysis	Information regarding antemortem assumption. Free of proteins and lipids, helpful for immunoassys tests. Not affected by postmortem redistribution	Wide detection window.No strong correlation between concentration and pharmacological consequences
Body organs (liver, brain, kidney)	Helpful to interpret blood concentration of the drug	Useful in case of lipophilic drugs and extended post-mortem interval. Kidney specimen could be helpful in case of heavy metal poisoning	Part of the liver (left lobe) may be more affected by post–mortem redistribution from the stomach. Brain concentration may change based on the region
Bile	Screening, to study drugs undergoing hepatic matabolism	Depot for substances and metabolites with biliary excretion	Influenced by hepatic metabolism and hepatic diseases
Gastric Content	Suspicious or autopsy evidence of oral drug assumption	An estimation of the amount of drug or poison present in the gastric volume is helpful to decide whether an analytical finding is rather more consistent with an overdose or a therapeutic dosage taken just prior to death	Small detection window, not useful in case of alternative route of administration
Hair	Chronic and previous use of substances evaluation (drug testing in workplace, crimes facilitated by drugs, abstinence monitoring, child custody)	Easy and non invasive collection of the sample, easy transportation and storage (no need of refrigeration), no time dependent (useful also in decomposed bodies), no risk of infection during collection, tolerance	No information regarding recent use and acute intoxication. Quantitative confirmatory techniques needed (GC-MS or LC-MS)
Oral fluid	Useful for drug intake monitoring and recent drug exposure (drivers)	Simple, safe, easy and non invasive collection, drug levels correspond to plasma levels. Helpful for recent assumption of psychoactive drugs	Influenced by age, gender, smoking or oral substance assumption, oral cavity environment. Small volume, very sensitive methods needed
Sweat	Used to test drug assumption in recovery centers, drug-addicted in rehab, in workplace	Non invasive collection, cumulative registration of substances, easy storage.	Not sensitive for many substances (such as THC), much lower sensitivity and specificity than urine for EMIT
Vitreous humor	Similar to blood and urine testing	Useful if traditional matrices are not available or inappropriate (burned or decomposed bodies). Less interference with environment and microbial activity (alcohol detection)	Less sensitive and specific for lipophilic substances. Drugs can reach vitreous humor only in free form, not if are bound to proteins
Breast milk	Used to investigate mother’s drug exposure and infant exposure to damaging substances	Short detection window	The detection rate of the substances depends on the characteristics (pKa, lipid solubility, pH, bound to protein)

## Data Availability

All data are included in the main text.
